# Antibiotic prophylaxis among Indian women undergoing cesarean sections

**DOI:** 10.6026/973206300210594

**Published:** 2025-04-30

**Authors:** Bhavani Kaveti, Gadiparthi Anusha, Priyadarshini Ramesh, Madhurika Jalakam, Prashannalakshmi A, Reema Reji, Yagvalkya Sharma

**Affiliations:** 1 Department of Obstetrics and Gynaecology, Sri Sai Ram Fertility and Women Health Care Hospital, Gaddiannaram, Hyderabad, Telangana, India; 2 Department of Obstetrics and Gynaecology, Vijay Marie Hospital, Veernagar, Saifabad Hyderabad, Telangana, India; 3 Department of Obstetrics & Gynecology, Madras Medical College, Chennai, India; 4 Department of ITU, Queen Elizabeth Queen Mother Hospital, East Kent Hospital University Foundation Trust, United Kingdom; 5 Department of Medicine, Tashkent Medical Academy, Urgench branch, Uzbekistan; 6 Department of Pediatrics, Dr. KM Cherian Institute of Medical Sciences, Umayattukkara Road, Kallissery P.O, Chengannur, Kerala, India; 7 Department of Research, Kalp Research Work, Mathura, Uttar Pradesh, India

**Keywords:** Cesarean section, antibiotic prophylaxis, surgical site infection, adherence to guideline, postoperative infection, tertiary care hospital

## Abstract

Cesarean sections are associated with a high risk of postoperative infections, making timely and appropriate antibiotic prophylaxis essential. Hence, a six-month study of 100 patients found 85% compliance with recommended guidelines, with SSIs (Surgical site infections) occurring in 3% of timely cases versus 9% in delayed cases. Deviations, such as delayed administration or inappropriate antibiotic use, modestly increased infection risk. Key factors in reducing SSIs were timely antibiotic delivery and appropriate selection. Improved training, surveillance and audits are recommended to enhance compliance so as to ensure safe obstetric surgical practices.

## Background:

A C-section is the most common surgical procedure in the world, which also happens to be on an increasing rate and it goes on steadily for medical reasons, obstetric reasons and patient-centered reasons. The list of indications for C-sections would include fetal distress, abnormal fetal presentations, a failure of labor to progress and previous cesarean deliveries among many others. Although a lifesaving procedure for mother and child, the C-section surgeries are not risk-free [1]. One of the most common complications after surgery is SSI, which raises deep concerns for the health of patients; extended days spent in a hospital and increased costs to healthcare [2]. Infections post C-section may vary from superficial wound infections up to more severe problems such as endometritis and deep abdominal infections with a chance of causing sepsis, elevating mortality and death. Now it is increasingly recognized that there is a substantial risk of infection during the delivery of a cesarean section and antibiotic prophylaxis has become a key component of perioperative management [3, 4]. The overall goal of prophylactic antibiotic therapy is to reduce bacterial burden at the surgical site, lowering the probability of subsequent infection and better patient outcome. However, prophylaxis effectiveness is dependent upon the timing of administration of the antibiotic and the type of antibiotic chosen, as well as intrinsic patient-specific risk factors for infection, such as obesity, diabetes and any prior history of infection [5]. Recommendations for this practice include that from the American College of Obstetricians and Gynecologists and the World Health Organization: a single dose of first-generation cephalosporin given within an hour before the incision site in an uncomplicated cesarean section [6]. This timing is highly essential since the peak level of antibiotics is expected to occur at the time when the exposure by the microorganisms will be maximally allowed, hence improving the effectiveness of the antibiotics. Though facilities may have compliance with the different standard guidelines recommended, they also vary between different facilities. Several authors have pointed out that the delay in the administration and use of broad-spectrum antibiotics without specific indications is associated with increased rates of postoperative infections [7]. Prophylactic antibiotics in tertiary care hospitals are usually hard to standardize due to the heavy volume of surgery and the complex conditions treated [8]. Variability in adherence may be due to several logistical barriers, inconsistency in the practices that occur during perioperative periods, or just a failure to standardize specific protocols for certain patient populations. In addition to preventing infection, strict adherence to protocols impacts antibiotic stewardship [9]. Misuse or overuse of antibiotics in surgical prophylaxis leads to the development of resistant bacteria. This is yet another obstacle that makes infection management difficult. Therefore, analysis of antibiotic prophylaxis practice in cesarean sections may lead to betterment of the outcomes of the patients as well as optimize the use of antibiotics while avoiding resistance [10]. Therefore, it is of interest to monitor the current antibiotic prophylaxis practices at the time of cesarean sections, focusing on adherence to recommended guidelines, timing, selection of antibiotics and outcome postoperatively in a high-volume tertiary care hospital.

## Materials and Methods:

## Study design and setting:

This was a prospective observational study conducted at the tertiary care hospital to assess the practices related to antibiotic prophylaxis used for cesarean sections. The study was undertaken in the Department of Obstetrics and Gynecology.

## Selection criteria:

The study included women scheduled for either elective or emergency cesarean sections, irrespective of indication.

## Exclusion criteria included:

[1] Women presenting with signs of active infection, such as fever or leukocytosis.

[2] Those already receiving antibiotic therapy prior to surgery.

[3] Patients with severe anemia or underlying medical illnesses.

[4] Women referred from other hospitals after labour onset.

A convenience sample of 100 participants meeting these criteria was selected to account for time constraints, with each participant providing written informed consent.

## Data sources and variables:

Data were gathered through direct observation and patient medical records, focusing on variables such as:

[1] Patient demographics: Age, obstetric history and relevant health status

[2] Surgical details: Type of cesarean section (elective or emergency) and surgical indications

[3] Antibiotic prophylaxis: Timing, type and dosage of prophylactic antibiotics administered.

[4] Postoperative outcomes: Incidence of infection, any adverse events related to antibiotic use and length of hospital stay. Participants were monitored through their hospital stay to capture postoperative outcomes until discharge.

## Statistical analysis:

To analyze, the data were entered into a standardized format. Several descriptive statistics captured patient demographics as well as the patterns of use of perioperative antibiotic practice. The proportion of cases using antibiotic prophylaxis as prescribed and the associations between prophylaxis timing and outcomes after surgery were calculated.

## Results:

Table 1 summarizes the Demographic and clinical characteristics of participants. The mean age among the 100 women who were to undergo cesarean section was 28.4 years ± 4.2 SD. Schedule elective operations constituted 60% of the patients. Emergency operations were conducted on the remaining 40%. The primary reasons for cesarean delivery included fetal distress that had taken up 25%, previous cesarean section 20% and failure to progress in labor 18%. Other obstetric or medical conditions, including many other factors, accounted for 37% of the indications for the procedure. This report outlines common causes of cesarean deliveries in this population and shines a light on the diversity of clinical conditions for which surgical prophylaxis is used. Table 2 reflects that though majority of the treatments had been administered following the protocol, an enormous proportion of the patients had unaligned prophylaxis in terms of time as well as choice of prophylaxis. The study provides first-hand information on the prophylaxis guidelines of antibiotics, which concerns the timing and choice of antibiotics. The timing of antibiotic prophylaxis indicates that 72% of the participants received the treatment within 30 to 60 minutes after the incision was performed on the skin and comply best with World Health Organization's recommendations. On the other hand, 28% received the antibiotics outside the recommended time of 30-60 minutes. Apart from this, 85% of women received first-generation cephalosporins, which go against guidelines, while 15% received alternative antibiotics. Table 3 this contributes post-surgical results regarding infection rates, the length of stay in hospitals and side effects against antibiotics. The incidence of postoperative infection was 12% and infections were significantly higher when the antibiotics were given at times other than that recommended, suggesting a time-associated relationship between the infections and the antibiotic administration time. The overall population means length of stay in the hospital was 4.8 ± 1.5 SD, while patients who manifested infections raised the average time in days to 6.3. Those patients who did not manifest infection spent an average of 4.3 days. There were only 4% of the patients that were treated with the administered antibiotics and showed a slight reaction to an antibiotic, while severe reaction was not noted. Such results may indicate that implementation of prophylactic recommendations is associated with advantageous outcomes after surgery, like a reduced incidence of postoperative infection and LOS.

## Discussion:

Modern SAP is a guideline for a prophylaxis to infection, especially for the patient with a cesarean section. Indeed, proper SAP timing and agent selection are the key elements in reducing the risk of infection as well as optimizing outcomes in patients because results of this study concordant with other literature which states that antibiotics given appropriately, at right times with the appropriate agents significantly reduce postoperative infections [11]. It has been noticed that SAP in cesarean techniques prevent two of the most common complications arising with cesarean delivery namely, endometritis and wound infections [12]. For example, health organizations have put down guidelines regarding the administering of antibiotics within one hour of the time of surgical incision to ensure tissue levels at the time of surgical incision [13-14]. Our results agree with such recommendations because these delays have been demonstrated to be associated with elevated risks for infection at the time this delay is incurred after the time of surgical incision [15]. Other studies have ascribed specific subpopulations of patients who are at elevated risks for infection following cesarean delivery, one of which includes patients with bacterial vaginosis. Although it is definitely associated with increased risk of endometritis, Caesarean delivery in a woman with bacterial vaginosis should also be preceded by such evaluation for risk factors and prophylactic antibiotics should also be provided [16, 17]. Much controversy still exists about what single antibiotic would suffice for the use in Caesarean prophylaxis. However, although the agent remaining the winner is cefazolin, clindamycin or metronidazole are offered in any case if the patient is allergic to beta-lactams [18-19]. Variability in the practice also fits well with the plea for better guidance and also with the scope of prophylaxis to be expanded for more ample coverage as some studies has pointed out [20]. The time of administration has come to be known to be the second one that is important as well [21]. There have been studies published that are based on just one study carried out in Uganda, where it has been documented that cases of infections were very few due to intravenous administration before pre-incision compared with cases due to intravenous administration following cord clamping [22]. This comes to validate the results documented by the existing procedures in contrast to the performance based on pre-incision delivery which showed a decrease of cases among those who were infected after compliance. Moreover, antibiotics disturb the normal flora of bacteria in particular on the endometrium and their outcome regarding recovery after delivery cannot be excluded. A summary of results in some of these review studies shows that preventive antibiotics outcome leads to decreased bacterial colonization within the endometrium thus positive implications for general outcomes in the postpartum stages even though further research needs to be done to better understand these implications. This has been proven not only at the patient-level effect but also to the field of antibiotic stewardship. This includes increased resistant strains; long-term effects associated with such an increase bring dire consequences to healthcare systems [23-24]. Hence, keeping them effective for their true prophylactic indications requires limiting unnecessary exposure through adherence to SAP guidelines. This is partly evidenced in that some antibiotics are documented not to cross the placental barrier in significant amounts after administered for prophylaxis, hence limiting such risk to the foetus. For example, colonization of amniotic fluid by Urea plasma urea-lyticum was proven to be associated with maternal infections, hence further supporting SAP's role in its own right to reduce risk in such a manner [25-26]. The minimum exposure to the foetus and effectiveness against pathogens should be weighed against choosing the right antibiotics in pregnant individuals. However, this study verifies an assumption that SAP is not applied uniformly, in the form of a standard requirement because of het- erogeneity in timing and selection techniques [27]. Other research indicates the same result: SAP was either on-time or SAP with the neglect of patients' individual peculiarities, such as allergy or a history of colonization with bacterial flora [28]. High-quality and focused education and surveillance should be available to support SAP adherence development in healthcare organisations [29]. In a study assessing risk factors associated with no prophylaxis found that forgetting or skipping due to time constraints was an important cause for not giving antibiotic. Having a quality improvement measures including clinical audit improves the uptake of standard guidelines [30].

## Strengths and limitations:

This is a prospective observational study that has some strengths and limitations adding to findings. In this regard, it is clear that a holistic data collection method was adopted-with diversified population and clinical characteristics-implemented in the study. This knowledge will provide better insight into how antibiotic prophylaxis is effective and appropriate for surgical procedures within that specified population. This was also a prospective design and thus reduced the risk of recall bias, further fortifying the validity of findings. This is, however, a study with some of the natural challenges of an observational study wherein one could not conclude definitely whether any association is causal. It was conducted in one tertiary care centre, thereby putting some limits to generalizability. Variability may also be evidenced among surgeons in clinical practice, thus potentially affecting the outcome of the findings to be presented by this study.

## Conclusion:

We show the critical role of proper antibiotic prophylaxis in preventing postoperative infections and underscore the need for strict adherence to established protocols to ensure patient safety and quality care. Continuous education and training of healthcare practitioners are paramount to optimizing antibiotic use in surgical practice. Further large-scale, multi-centric studies are essential to validate these findings and explore additional factors influencing the efficacy of antibiotic prophylaxis across various surgical procedures.

## Figures and Tables

**Table 1:** Patient demographics and surgical details

**Variable**	**Total (N = 100)**
Mean Age (± SD)	28.4 ± 4.2 years
Elective Cesarean Section	60 (60%)
Emergency Cesarean Section	40 (40%)
**Primary Indications**	
- Foetal Distress	25 (25%)
- Previous Cesarean Section	20 (20%)
- Failure to Progress	18 (18%)
- Other	37 (37%)

**Table 2:** Adherence to antibiotic prophylaxis practices

**Variable**	**Total (N = 100)**
Antibiotics Administered 30-60 mins Prior	72 (72%)
Antibiotics Administered Outside Window	28 (28%)
First-Generation Cephalosporins Used	85 (85%)
Other Antibiotics Used	15 (15%)

**Table 3:** Postoperative outcomes

**Outcome**	**Total (N = 100)**
Postoperative Infection	12 (12%)
Average Hospital Stay (days)	4.8 ± 1.5 days
Adverse Reactions to Antibiotics	4 (4%)
